# The BARIAlink Global Collaborative Platform: Just Another Educative Tool or a Pioneering Evolution in the Treatment of Metabolic and Obesity-Related Disease?

**DOI:** 10.1159/ofa/adzag001

**Published:** 2026-07-24

**Authors:** Ala Wafa, Jacques Himpens, Antonio Torres, Chetan Parmar, Bruno Dillemans

**Affiliations:** a Misurata University, Tripoli, Libya; b Bariatric-metabolic surgery department Delta-CHIREC hospital, Brussels, Belgium; c General and Digestive Surgery Service, Hospital Clínico San Carlos, Department of Surgery, Complutense University Medical School, Universidad Complutense de Madrid (UCM), IdISSC, Madrid, Spain; d Whittington Hospital, University College London, London, UK; e Department of General, Vascular and Pediatric Surgery, AZ Sint-Jan AV Brugge, Bruges, Belgium

**Keywords:** Bariatric surgery education, BARIAlink, Surgical complications management, Continuing medical education

Dear Editor,

Metabolic bariatric surgery (MBS) is widely recognized as one of the most effective interventions for severe obesity, yielding substantial and lasting weight loss, as well as the remission of type 2 diabetes and other associated comorbidities [1]. The global increase in the number of procedures demonstrates that the specialty’s focus is appropriately evolving from simply providing access to care to ensuring the quality and safety of that care [2].

Despite significant advancements in standardization and surgical techniques, complications such as anastomotic leaks, bleeding, and internal hernias persist as inherent risks. The learning curve for complex and revisional procedures remains notably steep, and the effective management of complications is a crucial skill often gained through challenging and sometimes isolating experiences [3, 4]. Complications vary in severity, chronology, and concomitant episodes, making it difficult to categorize them into a single comprehensive framework. Furthermore, the severity of complications is often hard to estimate without the input of experts. A surgeon faced with a rare complication on a weekend cannot afford to wait for the next conference or journal publication for guidance. This disconnection between the generation of knowledge and its practical application at the point of care constitutes a significant vulnerability in patient treatment pathways.

Traditional forms of continuing medical education, such as textbooks, academic journals, and annual conferences, while valuable, are hampered by limitations in timeliness, interactivity, and accessibility. The digital revolution presents a powerful solution to this problem. While tele-mentoring and virtual communities have shown promise in the surgical field [5, 6], many current platforms are either too general or serve as passive archives of recorded lectures, allowing little or no interaction. A clear need existed for a dedicated, interactive, and professionally moderated platform that specifically addresses the high-stakes challenges unique to MBS.

Responding to the widespread requests of the MBS community, the BARIAlink platform was conceptualized in 2016 under the leadership of Dr. Bruno Dillemans. It was envisioned not merely as a webinar service but rather as a comprehensive digital ecosystem – a “digital coliseum” – where bariatric professionals can convene to share, debate, and learn from their most challenging cases in a structured and expert-led setting. BARIAlink is an IFSO-endorsed platform that has been actively presented at multiple world IFSO congresses, including those in London, Madrid, and Napoli, with dedicated sessions.

The theoretical underpinning of BARIAlink is the concept of “Communities of Practice” (CoPs), as articulated by Wenger and Snyder [7]. A CoP is defined as a group of individuals who share a common interest or passion and who learn to improve their practice through regular interaction. BARIAlink has operationalized this concept on a global scale for the bariatric surgery community. The platform’s architecture is founded on two synergistic pillars: a collaborative community pillar and a structured educational pillar.

The collaborative community pillar constitutes the vibrant core of the platform. It is a secure, private online forum accessible to a verified community of bariatric surgeons, endoscopists, and other members of the multidisciplinary team. This community functions primarily through the submission and discussion of complex, anonymized cases. These comprehensive presentations include patient history, operative notes, imaging studies, endoscopic findings, and laboratory data. The subsequent threaded discussions facilitate in-depth, multi-perspective dialogues on diagnostic challenges, technical nuances of re-interventions, and long-term management strategies.

To complement the organic, peer-to-peer learning, BARIAlink incorporates a formal curriculum delivered through its virtual classrooms. These are live, interactive sessions led by a global faculty of recognized experts in specific sub-specialties, such as revisional surgery, the management of leaks, or metabolic outcomes. The format is intentionally Socratic, involving a concise expert lecture followed by the presentation of complex cases. The expert moderator, in conjunction with the live audience, guides the discussion, challenging assumptions and presenting evidence-based alternatives. This approach transcends passive knowledge transfer, fostering active cognitive training that is essential for developing sound surgical judgment.

A cornerstone of the BARIAlink ecosystem is its comprehensive library, which provides members with on-demand access to the platform’s entire archive of discussed cases and structured educational sessions. This powerful resource allows surgeons to readily search for and learn from specific topics, complications, or surgical techniques, transforming the platform from a real-time discussion forum into a lasting educational repository. Members can review multiple expert approaches to a rare complication before encountering it in their own practice, significantly enhancing their preparedness and decision-making capabilities.

The BARIAlink model directly addresses several well-documented challenges in surgical education and patient safety. By providing vicarious experience through exposure to numerous complex scenarios, the platform helps flatten the learning curve associated with managing rare complications. This aligns with the educational theory of “deliberate practice,” which posits that performance improves through repeated exposure to challenging scenarios with immediate feedback [8]. Furthermore, by disseminating management strategies from leading international experts, BARIAlink serves as a powerful catalyst for the standardization of care. Discussing cases in a group setting also helps mitigate individual cognitive biases by exposing surgeons to multiple differential diagnoses and thought processes [9].

In conclusion, the BARIAlink platform represents a pioneering and highly effective response to the evolving educational needs of the global bariatric surgical community. By successfully creating a vibrant digital CoP centered on rigorous case discussion and expert-led virtual classrooms, it has established a new paradigm for continuous professional development. It transforms surgical education from a periodic, passive event into a continuous, highly dynamic, and active process. In doing so, it serves as an indispensable tool for standardizing high-quality care, flattening the learning curve for complex problem management, and safeguarding patient outcomes on a worldwide scale. As digital technology continues to evolve, BARIAlink provides a replicable model for other surgical specialties to emulate.

## Conflict of Interest Statement

Prof. Dr. Antonio Torres was a member of the journal’s Editorial Board at the time of submission. The authors declare that there are no other conflicts of interest for this study.

## Funding Sources

The authors did not receive any funding for this work.

## Author Contributions

Ala Wafa wrote substantial portions of the original draft, while Jacques Himpens, Antonio Torres, Chetan Parmar, and Bruno Dillemans reviewed and edited, providing significant feedback on the manuscript.
